# Translation, adaptation, and validation of the Tolerance of Ambiguity in Medical Students and Doctors (TAMSAD) scale for use in Japan

**DOI:** 10.1186/s12909-023-04391-1

**Published:** 2023-06-05

**Authors:** Hirohisa Fujikawa, Daisuke Son, Mikio Hayashi, Kayo Kondo, Masato Eto

**Affiliations:** 1 Center for General Medicine Education, School of Medicine, Keio University, 35 Shinanomachi, Shinjuku-ku, Tokyo, 160-8582 Japan; 2 Department of Medical Education Studies, International Research Center for Medical Education, Graduate School of Medicine, The University of Tokyo, Bunkyo-ku, Tokyo, Japan; 3 Department of Community-based Family Medicine, Faculty of Medicine, Tottori University, Yonago, Tottori Japan; 4 Center for Health Professions Education, Kansai Medical University, Osaka, Japan; 5 Master of Medical Sciences in Medical Education, Harvard Medical School, Boston, MA USA; 6 School of Modern Languages and Cultures, Durham University, Durham, UK

**Keywords:** Tolerance of ambiguity, Tolerance for ambiguity, Uncertainty, Factor analysis, Medical students, Medical trainees, Residents

## Abstract

**Background:**

Ambiguity is inherent to the medical field; hence, assessing and educating medical trainees regarding ambiguity tolerance is essential. The Tolerance of Ambiguity in Medical Students and Doctors (TAMSAD) scale—a novel instrument that assesses ambiguity tolerance in clinical settings—has been widely used for medical education research in Western countries. However, a version of this scale applicable to the intricate clinical contexts of Japan has not yet been developed. In this study, we developed the Japanese version of the TAMSAD (J-TAMSAD) scale and tested its psychometric properties.

**Methods:**

In this multicenter study, we collected data through a cross-sectional survey in two universities (medical students) and ten hospitals (residents) across Japan, and evaluated the structural validity, criterion-related validity, and internal consistency reliability of the J-TAMSAD scale.

**Results:**

We analyzed the data of 247 participants. The sample was randomly divided in half, with exploratory factor analysis (EFA) performed on one half and confirmatory factor analysis (CFA) on the other. EFA led to an 18-item J-TAMSAD scale comprising five factors. CFA showed acceptable fit for this five-factor model (comparative fit index = 0.900, root mean square error of approximation = 0.050, standardized root mean square residual = 0.069, goodness of fit index = 0.987). There was a positive correlation between the J-TAMSAD scale scores and total reverse scores on the Japanese version of the Short Intolerance of Uncertainty Scale (Pearson correlation coefficient 0.41). The internal consistency was found to be satisfactory (Cronbach’s alpha 0.70).

**Conclusions:**

The J-TAMSAD scale was developed, and its psychometric properties were confirmed. The instrument can be useful for assessing tolerance of ambiguity among medical trainees in Japan. With further validation, it could be used to verify the educational effectiveness of curricula that foster ambiguity tolerance in medical trainees, or even in research assessing the relationship with other variables.

**Supplementary Information:**

The online version contains supplementary material available at 10.1186/s12909-023-04391-1.

## Background

The notion of ambiguity is inherent to medicine practice because of knowledge limitations, uncertainty of diagnosis, therapy, and outcomes as well as patient response unpredictability [1]. Various studies have indicated that lower Tolerance of Ambiguity (TOA) precipitates a range of outcomes, such as reduced psychological well-being [2, 3], increased odds of burnout [4], and greater negative attitudes toward underrepresented patients [5]. Therefore, it is crucial to nurture medical learners’ TOA; thus, assessment tools for this variable are warranted for effective TOA education.

To date, various instruments for measuring TOA have been developed, with the most widely used tool being the 16-item Likert-type scale of tolerance-intolerance of ambiguity devised by Budner [6] and its variants. Recently, Geller et al. developed the Tolerance for Ambiguity Scale, which has been included in the Association of American Medical Colleges Matriculating Student Questionnaire [7]. These scales, however, are neither clinically contextualized nor assessed in the specific context, such as undergraduate or postgraduate medical education [8]. Thus, for a considerable period, there had been a pertinent need to develop a tool that could measure TOA in clinical contexts.

Then, in 2015, Hancock et al. developed the Tolerance of Ambiguity in Medical Students and Doctors (TAMSAD) scale in the UK [8]. They developed the questionnaire after a thorough literature review of existing scales and expert consultation. It was well validated through psychometric analyses of survey data from approximately 500 medical students and postgraduate trainees, consequently forming a 29-item scale. Since the TAMSAD scale is the only tool that specifically assesses TOA in clinical situations, it has been widely used for medical education research in Western countries [2, 9, 10].

However, to our knowledge, there are no available instruments for TOA for clinical contexts in Japan, and this gap can hinder TOA education in the country. Accordingly, the development of such instruments could help further inform and enhance TOA education for Japanese learners and trainees. In this study, we developed the Japanese version of the TAMSAD (J-TAMSAD) scale and examined its validity and reliability.

## Methods

### Design, setting, and participants

To better understand the Japanese context, here we briefly describe the medical education curriculum in Japan. The curriculum lasts six years [11]: in the first and second years, medical students study liberal arts and basic medicine (e.g., anatomy, biochemistry, and physiology); in the third and fourth years, they study clinical medicine, mainly through classroom lectures. To be eligible for subsequent clinical practice, medical students must pass Computer-Based Testing and Objective Structured Clinical Examination. After passing these examinations, medical students are awarded the title of “student doctor” and then proceed to clinical practice, which lasts approximately two years. In this study, given the context of the medical education system in Japan, we excluded first- through fourth-year medical students because they rarely engage in clinical practice, making it difficult for them to respond to the TAMSAD scale, a measure of ambiguity tolerance in clinical settings.

In July 2022, two universities and ten hospitals—varying in location, size, and type (Table 1)—participated in this cross-sectional study. We used SurveyMonkey (www.surveymonkey.com) to distribute our survey and collect responses. A web link was created, and an invitation email was sent to clinical year medical students (i.e., fifth- and sixth-year medical students) enrolled in these participating universities and the residents of the hospitals. They were informed that participating in this study would be voluntary and that refusing to participate would not result in any disadvantages, and they were asked to complete an online questionnaire. Non-respondents were reminded to complete the survey thrice via email. As the questionnaire was only accessible via the web link, it was a closed survey.

**Table 1:** Characteristics of the participating institutions

	N (%)
**University (Medical students)**	
University types	
National	1 (50)
Private	1 (50)
University locations	
Kanto	1 (50)
Kinki	1 (50)
**Hospitals (Residents)**	
Hospital types	
Community hospital	8 (80)
University hospital	2 (20)
Hospital locations	
Hokkaido and Tohoku	2 (20)
Kanto	2 (20)
Chubu	2 (20)
Kinki	1 (10)
Chugoku and Shikoku	2 (20)
Kyushu	1 (10)
Hospital sizes	
≤400 beds	2 (20)
401–600 beds	4 (40)
≥601 beds	4 (40)

### Measures

#### Original TAMSAD scale

In the original study of the TAMSAD scale [8], factor analysis failed to find any simple solution, meaning that its 29 items could not be subdivided into interpretable factors and it was defined as a unidimensional tool. Respondents answered each questionnaire item using a 5-point Likert scale from 1 to 5 (1 = *strongly disagree*; 5 = *strongly agree*). After averaging the responses of the 29 items, the resulting scores were linearly transformed to a scale of 0–100 using the following formula: “transformed score = 25*(average score − 1)” [8]. Higher scores indicated greater TOA.

### Procedure for translation

We reached out to the original developer of the TAMSAD scale by e-mail to request permission to translate the scale into Japanese, and their agreement was obtained. In compliance with the cross-cultural adaptation guidelines suggested by Beaton et al. [12], the original TAMSAD scale items in English were translated into Japanese. The translation process was as follows:

1. Forward translation: Three translators (the authors: HF, DS, and KK), all of whom had experience in questionnaire translation in the medical education field [13, 14], independently conducted forward translations.
2. Synthesis: The drafts of each translator were compared item by item, and the translations were synthesized by the three translators (Ver. 1).
3. Back-translation: Ver. 1 was translated back into English by professional bilingual translators who were not involved in the study and, therefore, had no prior knowledge of the scale. The three translators, thereafter, compared the back-translated version with the original English version and proofread it to produce Ver. 2.
4. Expert review: We asked a medical education expert (MH) to review Ver. 2 and, thereafter, modified it based on the feedback (Ver. 3).
5. Feedback from the original scale developer: We contacted the original English scale developer by e-mail and asked him to review Ver. 3. We received feedback from him and made further revisions accordingly (Ver. 4).
6. Pre-testing: We performed a pilot test with three trainees (two residents and one medical student), who were interviewed on whether Ver. 4 exhibited expressional clarity and meaning.

Pilot testing showed no problematic items during the translation process; therefore Ver. 4 was selected as the final version. The tool’s face and content validity was confirmed by all the authors.

### Statistical analysis

We tested the structural validity of the J-TAMSAD scale using both exploratory and confirmatory factor analysis (EFA and CFA). Considering the problems with performing both EFA and CFA on the same sample [15], we split the sample into two independent groups randomly—a process also known as split-half validation analysis.

To verify the suitability of the data set for conducting EFA, we first checked the Kaiser–Meyer–Olkin (KMO) measure of sampling adequacy and Bartlett’s test of sphericity. To perform EFA, a KMO value of 0.60 or higher and a significant Bartlett’s sphericity test are recommended [16]. Then, we performed maximum likelihood EFA with promax rotation. We conducted EFA to ensure that the developed scale is optimized for the Japanese culture and society. Additionally, this procedure helped us remove problematic items within the context of the Japanese healthcare setting before conducting CFA. We consulted the results of the parallel analysis to determine the number of factors [17]. Next, we excluded those items that had a factor loading below 0.30 or cross loadings of less than a 0.15 difference from an item’s greatest factor loading.

We conducted maximum likelihood CFA to assess model fitness. We evaluated two models: the five-factor model specified by EFA (Model A) and the original one-factor model (Model B). The following indices were used: comparative fit index (CFI), root mean square error of approximation (RMSEA), standardized root mean square residual (SRMR), and goodness of fit index (GFI). According to the guidelines for related cut-off values [16, 18, 19], models with CFI and GFI close to 0.90 or greater and RMSEA and SRMR close to 0.08 or less are considered to provide an acceptable fit to the data.

To examine criterion-related validity, we used the total scores for the J-TAMSAD scale and the total reverse scores for the Japanese version of the Short Intolerance of Uncertainty Scale (J-SIUS). Uncertainty is an overarching and superordinate construct, being the response to either ambiguity, probability, or complexity; by contrast, ambiguity is a subordinate construct—a primary source of uncertainty, and a property of information associated with the lack of reliable, credible, or adequate information [20]. Thus, uncertainty is apparently similar to ambiguity, but is, in fact, conceptually distinct from ambiguity [21]. Therefore, we deemed that the reverse scores of the J-SIUS, which measures intolerance of uncertainty, would be useful for examining criterion-related validity.

The IUS is a well-validated instrument comprising 27 items that measure intolerance of uncertainty [22]. Carleton et al. developed the SIUS based on this scale [23], which was, reportedly, highly correlated with the original IUS and demonstrated good reliability and validity [23, 24]. The 12-item SIUS is a relatively simple instrument with all items answered on a Likert scale of 5-point response options (1: *not at all characteristic of me* to 5: *entirely characteristic of me*), making it highly convenient for both research and clinical use [25]. The J-SIUS is the translated version of the SIUS and, reportedly, exhibits high reliability and validity [25]. Since our study endeavored to develop a “tolerance” (not “intolerance”) scale for ambiguity, all J-SIUS items were reverse-coded (e.g., the 1 score was changed to 5) [26]. We summed them up to confirm the criterion-related validity, and used Pearson correlation coefficients to check whether J-TAMSAD scores predicted the total reverse scores of the J-SIUS. If correlation coefficients exceeded 0.30, they were considered meaningful [27].

We calculated Cronbach’s alpha coefficients using data from the whole sample to examine internal consistency reliability. Cronbach’s alpha values are characterized as follows: < 0.50, insufficient; 0.50–0.69, moderate; 0.70–0.79, satisfactory; and ≥ 0.80, good [28].

Finally, we conducted descriptive statistics for the J-TAMSAD scores. We performed independent t-test and analysis of variance to explore the possible influence on the TAMSAD score of participants’ year group and gender. Due to the small amount of missing data, we decided to choose complete case analysis. We used R version 4.2.1 for all data analyses—psych package version 2.2.5 and GPArotation package version 2022.4-1 for EFA [29, 30], and lavaan package version 0.6–12 and semPlot package version 1.1.6 for CFA [31, 32].

### Ethical considerations

We asked participants to check the consent box at the beginning of our questionnaire to indicate their consent to participate in our study. Participants were enrolled into a drawing for one of ten ¥5,000 gift cards. We obtained ethical clearance from the Institutional Review Board of the University of Tokyo (2022010NI).

## Results

**Fig. 1:**
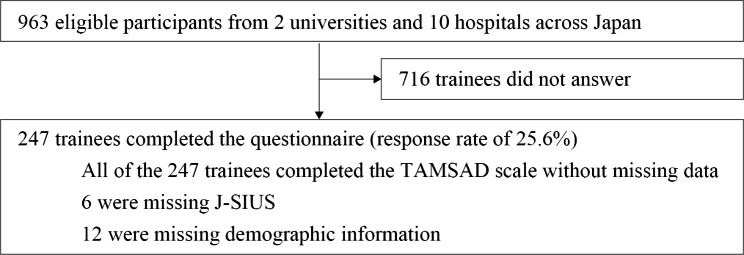
Sample flowchart in a study for the translation, cultural adaptation, and validation of the Tolerance of Ambiguity in Medical Students and Doctors (TAMSAD) scale to the Japanese setting. J-SIUS, Japanese version of the Short Intolerance of Uncertainty Scale

The questionnaire was completed by 247 (25.6%) of the 963 eligible participants. Figure 1 shows the participants’ flowchart and Table 2 depicts the respondents’ characteristics.

**Table 2:** Characteristics of the participants (N = 247)

Characteristic	N (%)
Gender	
Female	98 (39.7)
Male	137 (55.5)
Data missing	12 (4.9)
Stage/year of training	
Undergraduate (for medical students)	
Fifth-year	46 (18.6)
Sixth-year	54 (21.9)
Data missing	5 (2.0)
Postgraduate (for residents)	
Postgraduate years 1	65 (26.3)
Postgraduate years 2	70 (28.3)
Data missing	7 (2.8)

### Validity testing

We performed EFA on approximately half of the sample (n = 123). The KMO value was 0.7 (> 0.60), and Bartlett’s test of sphericity was significant (χ^2^ = 575.3236, df = 153, p < 0.001). Accordingly, we conducted EFA; after several iterations, 11 of the 29 items were omitted due to low factor loadings or cross-loadings, thus leading to 18 remaining items. The 11 items suggested for exclusion as a result of the EFA were reviewed by the research group, and it was decided to exclude them as they did not seem appropriate for the Japanese context. The final solution comprised the following five factors: 6 items were loaded on Factor 1 (which was named “Likes complicated, challenging, and vague situations in medical practice”), 3 items on Factor 2 (“Likes the mystery that is medicine”), 2 items on Factor 3 (“Tolerance for medical settings without single solution”), 3 items on Factor 4 (“Tolerance for things that are not black or white in medicine”), and 4 items on Factor 5 (“Tolerance for controversial circumstances in medical practice”). This process resulted in an 18-item J-TAMSAD questionnaire. The five factors accounted for 44.9% of the total variance (Table 3).

**Fig. 2:**
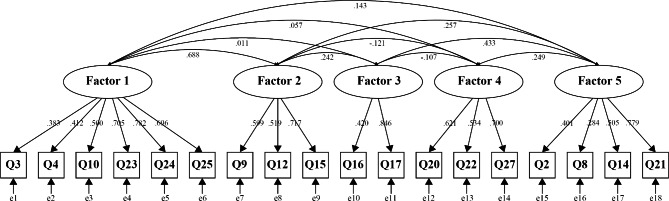
Factor structure of the Japanese version of the Tolerance of Ambiguity in Medical Students and Doctors (TAMSAD) scale (confirmatory factor analysis). Ellipses are latent variables (factors). Squares are observed variables (items). Values on single-headed arrows are standardized factor loadings. Values on double-headed arrows are correlation coefficients

For the other approximate half of the sample (n = 124), we performed CFA to compare the model fitness between the Model A (five-factor model) and Model B (the original one-factor model). In Table 4, the results of goodness of fit are shown. While the Model B did not converge, the Model A fit the data well (CFI = 0.900; RMSEA = 0.050; SRMR = 0.069; GFI = 0.987). Accordingly, we decided to adopt a five-factor model. Figure 2 indicates the path diagram of the CFA of the five-factor model. Prior research has suggested that ambiguity tolerance is composed of multiple components and that both positive and negative factors are present [33, 34]. Therefore, we considered the five-factor structure of our Japanese version to be theoretically valid.

Finally, we calculated the Pearson correlation coefficient of the J-TAMSAD scores and the total reverse scores of the J-SIUS. According to the original TAMSAD scale, we calculated the J-TAMSAD scores using the following formula to convert the scores to a scale of 0–100: “J-TAMSAD score = 25*(average score of the 18 items − 1).” The value of the Pearson correlation coefficient was 0.41, exceeding the threshold value of 0.30 (p < 0.001).

### Reliability testing and descriptive statistics

The Cronbach’s alpha values for all 18 items were 0.70, thus fulfilling the criteria for satisfactory internal consistency. The Cronbach’s alpha for Factors 1, 2, 3, 4, and 5 were 0.75 (satisfactory), 0.71 (satisfactory), 0.54 (moderate), 0.60 (moderate), and 0.52 (moderate), respectively. The descriptive characteristics of the J-TAMSAD scale are presented in Table 5. Table 6 shows the mean difference of the TAMSAD scale score between groups. There were no statistically significant mean differences by gender or year group. This process yielded the final version of the J-TAMSAD scale (Additional file).

**Table 3:** Results of exploratory factor analysis of the 18-item Japanese version of the Tolerance of Ambiguity in Medical Students and Doctors scale (N = 123)

Items (as in original version)	Factor loading				
	1	2	3	4	5
Q24. It is more interesting to tackle a complicated clinical problem that to solve a simple one	**0.996**	-0.214	-0.093	0.014	0.176
Q23. I like the challenge of being thrown in the deep end with different medical situations	**0.809**	0.040	-0.048	-0.104	0.213
Q25. I enjoy the process of working with a complex clinical problem and making it more manageable	**0.779**	-0.013	-0.096	-0.055	0.099
Q10. A patient with multiple diseases would make a doctor’s job more interesting	**0.427**	0.083	0.031	-0.085	-0.060
Q4. A good clinical teacher is one who challenges your way of looking at clinical problems	**0.398**	0.015	0.064	0.120	-0.242
Q3. I would be comfortable if a clinical teacher set me a vague assignment or task	**0.349**	0.168	0.112	0.088	-0.167
Q15. I like the mystery that there are some things in medicine we’ll never know	-0.041	**0.810**	-0.077	0.065	0.166
Q12. The unpredictability of a patient’s response to medication would bring welcome complexity to a doctor’s role	0.051	**0.698**	-0.087	-0.076	-0.069
Q9. I feel comfortable that in medicine there is often no right or wrong answer	-0.013	**0.589**	0.066	0.008	0.011
Q17. I find it frustrating when I can’t find the answer to a clinical question*	-0.208	0.143	**1.018**	-0.261	-0.027
Q16. Variation between individual patients is a frustrating aspect of medicine*	0.049	-0.106	**0.528**	-0.026	-0.027
Q27. To me, medicine is black and white*	-0.046	0.006	-0.171	**0.899**	-0.090
Q22. There is really no such thing as a clinical problem that can’t be solved*	-0.002	0.004	-0.082	**0.513**	0.003
Q20. No matter how complicated the situation, a good doctor will be able to arrive at a yes or no answer*	-0.076	-0.010	0.181	**0.415**	0.252
Q8. I think in medicine it is important to know exactly what you are talking about at all times*	-0.010	-0.026	-0.123	-0.105	**0.592**
Q21. I feel uncomfortable when textbooks or experts are factually incorrect*	0.045	0.193	-0.064	0.046	**0.541**
Q2. I have a lot of respect for consultants who always come up with a definite answer*	0.069	-0.193	0.146	0.062	**0.434**
Q14. Being confronted with contradictory evidence in clinical practice makes me feel uncomfortable*	0.039	0.180	0.174	0.037	**0.347**
	**Value**				
**Eigenvalue**	3.05	1.49	0.74	0.59	0.40
**Percentage variance explained**	14.5	9.2	7.7	7.2	6.4

* These were reverse items

**Table 4:** Confirmatory factor analysis of the Japanese version of the Tolerance of Ambiguity in Medical Students and Doctors scale (N = 124)

	CFI	RMSEA	SRMR	GFI
Model A (five-factor model)	0.900	0.050	0.069	0.987
Model B (one-factor model)	Did not converge			

Abbreviations: *CFI* comparative fit index, *RMSEA* root mean square error of approximation, *SRMR* standardized root mean square residual, *GFI* goodness of fit index

**Table 5:** Score distribution of the Japanese version of the Tolerance of Ambiguity in Medical Students and Doctors scale (N = 247)

Number of items	Mean	Standard deviation	Observed range	Skewness	Kurtosis
18	49.81	9.37	16.67–79.17	-0.069	0.824

**Table 6:** The mean difference of the Tolerance of Ambiguity in Medical Students and Doctors scale score between different groups

	Difference in TAMSAD scale score	95% CI	p value
Gender			
Female	-1.53	-3.96 to 0.89	0.214
Male	Reference category		
Year group			
5th-year medical students	-2.10	-7.05 to 2.86	0.701
6th-year medical students	1.17	-3.56 to 5.89	0.923
PGY1	1.77	-2.73 to 6.27	0.746
PGY2	Reference category		

Abbreviations: *CI* Confidence interval, *PGY* Postgraduate year, *TAMSAD* Tolerance of Ambiguity in Medical Students and Doctors

## Discussion

The original TAMSAD scale developed in the UK was translated and culturally adapted into Japanese in our study. We verified its good internal consistency reliability and structural and criterion-related validity. As far as we recognize, the J-TAMSAD scale is the first validated scale for assessing TOA among medical trainees in Japan.

We extracted five factors that emerged in this study from Japanese settings. Contrarily, the original TAMSAD scale is a unidimensional tool, with its factor analysis having failed to extract interpretable factors, for which we posit four possible reasons. First, TOA may be a multidimensional construct. Burdner classified ambiguous situations into the following three categories: novel, complicated, and insoluble situations [6]. Accepting unpredictable events as part of life, attempting to take direct action even when the outcome is unclear, and being able to work through incomplete information are characteristics that can be clubbed together as TOA [35, 36]. Thus, this concept inherently includes various elements, making it somewhat normal for the developed Japanese scale to be multidimensional. Second, it is possible that the factor analysis in the original English version of Hancock et al. was not performed properly. In their study, the authors used the Kaiser–Guttman criterion and scree plots to determine the number of factors, rather than the more robust methods often used today (e.g., parallel analysis). Third, it is possible that differences in demographics of the participants may have influenced the results. For medical students, our study included only grades 5–6, whereas the UK study included grades 1–5. It is questionable whether it was appropriate to involve medical students in the lower years of medical school, who would not have been familiar with the clinical context in the original UK study. Fourth, cultural and societal differences may exist in attitudes toward ambiguity. According to the cultural dimensions proposed by Hofstede [37, 38], the level of uncertainty avoidance differs by culture. Members of uncertainty-averse cultures tend to try to minimize the possibility of unstructured (i.e., new, unknown, surprising, and unusual) situations; these two cited studies also demonstrate that while Japan is a country that avoids uncertainty the most, the UK exhibits an extremely low uncertainty avoidance index score [37, 38]. The cultural differences between Japan and the UK will also probably be observed in different attitudes toward ambiguity in various aspects, such as preferences for structure, rules, risks, and decision-making. Such differences in attitudes may have influenced the study participants’ responses to the TAMSAD scale questionnaire, resulting in differences in the factor structure of the original English and Japanese versions of the questionnaire.

We believe that a strength of our study lies in the robustness of its translation methodology. Particularly, we translated the English questionnaire into Japanese following the recommendations of the cross-cultural instrument adaptation guidelines proposed by Beaton et al., which have been used worldwide [12]. We accordingly deem that the validity of our questionnaire was strengthened by this robust translation procedure. Furthermore, our study yielded an interesting finding from the factor analysis: two factors (Factor 1 and Factor 2) associated with a positive orientation toward ambiguity [33, 34], which is a concept often addressed negatively in the context of medical education [2]. In fact, this finding seems to be consistent with previous studies in Japan, which have shown that attitudes toward ambiguity consist of multiple aspects, including the following two positive elements: enjoyment and reception [34]. However, the paucity of research on the positive aspects of TOA remains, warranting further research on this topic.

### Implications

This study delivers the first Japanese version of the TAMSAD scale, which can be used as a novel instrument for measuring TOA in clinical settings across Japan. This tool may be helpful for assessing this construct in both undergraduate and postgraduate trainees and delivering education for them according to assessment results. Moreover, using this scale longitudinally from the time of students’ entry into medical school to their post-graduation career would surely yield interesting data. We also see value in potential explorations on the association between the TAMSAD scale and instruments for other variables (e.g., personality traits, professionalism, and specialty choice). The development of different language versions for this scale would certainly be valuable in deepening the international knowledge of TOA in clinical contexts.

### Limitations

Finally, this study has a couple of limitations. First, our sample size and response rate were relatively low. The COVID-19 pandemic is likely to have led most, if not all, potential participants to become greatly involved and focused on their work, consequently preventing them from securing time to complete the questionnaire. Second, other psychometric properties (e.g., convergent validity and test-retest reliability) have not been verified, though we tested the structural and criterion-related validity and internal consistency. In future studies, researchers could validate these psychometric properties of this instrument.

## Conclusions

Translating the TAMSAD scale into Japanese, we conducted an examination of its structural validity, criterion-related validity, and internal consistency reliability. We found that the instrument was useful in assessing medical trainees’ TOA in clinical contexts. Further validation studies can be conducted to verify the scale’s usability for testing the educational effectiveness of curricula that foster TOA in medical trainees, and for research examining the relationship of the scale with other variables.

## Electronic supplementary material

Below is the link to the electronic supplementary material.

Supplementary Material 1

## Data Availability

The corresponding author can provide the data sets generated and analyzed in this study upon reasonable request.

## Abbreviations

CFA: Confirmatory factor analysis
CFI: Comparative fit index
EFA: Exploratory factor analysis
GFI: Goodness of fit index
J-TAMSAD scale: Japanese version of the Tolerance of Ambiguity in Medical Students and Doctors scale
KMO: Kaiser–Meyer–Olkin
RMSEA: Root mean square error of approximation
SRMR: Standardized root mean square residual
TAMSAD scale: Tolerance of Ambiguity in Medical Students and Doctors scale
