# Supplementary material for: Translation, adaptation, and validation of the Tolerance of Ambiguity in Medical Students and Doctors (TAMSAD) scale for use in Japan
Source: BMC Med Educ. 2023 Jun 5;23:405. doi: 10.1186/s12909-023-04391-1 (PMC10240119; doi:10.1186/s12909-023-04391-1)
Supplement: Supplementary file 1 — Supplementary Material 1 [file 12909_2023_4391_MOESM1_ESM.docx]

The Japanese version of the Tolerance of Ambiguity in Medical Students and Doctors (TAMSAD) scale

| No. | English version | Japanese version | Was the item included in the final 18 item scale? |
| --- | --- | --- | --- |
| 1 | I would enjoy tailoring treatments to individual patient problems | 私は喜んで個々の患者の問題に合わせた治療を行うだろう | No |
| 2 | I have a lot of respect for consultants who always come up with a definite answer* | 私は、常に明確な答えを導き出す指導医を非常に尊敬している* | Yes |
| 3 | I would be comfortable if a clinical teacher set me a vague assignment or task | 私は、臨床の先生から漠然とした課題や仕事を課されても心地良く感じるだろう | Yes |
| 4 | A good clinical teacher is one who challenges your way of looking at clinical problems | 良い臨床の先生とは、臨床の問題に対する私の見方に疑問を投げかける先生のことである | Yes |
| 5 | What we are used to is always preferable to what is unfamiliar* | 慣れ親しんでいることは、慣れていないことよりも常に好ましい* | No |
| 6 | I feel uncomfortable when people claim that something is ‘absolutely certain’ in medicine | 医学において何かが「絶対確実」だと人が主張していると、私は違和感を覚える | No |
| 7 | A doctor who leads an even, regular work life with few surprises, really has a lot to be grateful for* | 驚きの少ない平凡で規則正しい仕事生活を送っている医師には、感謝すべきことが本当にたくさんある* | No |
| 8 | I think in medicine it is important to know exactly what you are talking about at all times* | 私は、医学では、我々が今何について話しているのかを常に正確に知っていることが重要だと思う* | Yes |
| 9 | I feel comfortable that in medicine there is often no right or wrong answer | 私は、医学では正解も不正解もないことが多いことを心地良く感じる | Yes |
| 10 | A patient with multiple diseases would make a doctor’s job more interesting | 複数の疾患を持つ患者がいると、医師の仕事はより興味深いものになるだろう | Yes |
| 11 | I am uncomfortable that a lack of medical knowledge about some diseases means we can’t help some patients* | 疾患によっては医学的知識が不足しているがゆえに助けられない患者がいることに、私は違和感を覚える* | No |
| 12 | The unpredictability of a patient’s response to medication would bring welcome complexity to a doctor’s role | 薬物療法に対する患者の反応が予測できないことは、医師の役割に喜ばしい複雑さをもたらすだろう | Yes |
| 13 | It is important to appear knowledgeable to patients at all times* | 常に患者に知識があるように見せることが重要である* | No |
| 14 | Being confronted with contradictory evidence in clinical practice makes me feel uncomfortable* | 私は、臨床現場で矛盾するエビデンスを突きつけられると、違和感を覚える* | Yes |
| 15 | I like the mystery that there are some things in medicine we’ll never know | 私は、医学には決して知ることのできないことがあるという不可解さが好きである | Yes |
| 16 | Variation between individual patients is a frustrating aspect of medicine* | 個々の患者の間にばらつきがあるのが、医学のもどかしい側面である* | Yes |
| 17 | I find it frustrating when I can’t find the answer to a clinical question* | 私は、臨床上の疑問に答えが見つからないときはもどかしいと思う* | Yes |
| 18 | I am apprehensive when faced with a new clinical situation or problem* | 私は、新しい臨床状況や問題に直面したときに不安になる* | No |
| 19 | I feel uncomfortable knowing that many of our most important clinical decisions are based upon insufficient information* | 私は、最も重要な臨床上の判断の多くが、不十分な情報に基づいていることを知ると、違和感を覚える* | No |
| 20 | No matter how complicated the situation, a good doctor will be able to arrive at a yes or no answer* | どんなに複雑な状況でも、良い医師はイエスかノーの答えに達することができるだろう* | Yes |
| 21 | I feel uncomfortable when textbooks or experts are factually incorrect* | 私は、教科書や専門家が事実と異なっていると、違和感を覚える* | Yes |
| 22 | There is really no such thing as a clinical problem that can’t be solved* | 解決できない臨床上の問題というのは存在しない* | Yes |
| 23 | I like the challenge of being thrown in the deep end with different medical situations | 私は、様々な難しい医療状況のなかに急に投げこまれるという難題が好きだ | Yes |
| 24 | It is more interesting to tackle a complicated clinical problem that to solve a simple one | 単純な臨床問題よりも、複雑な臨床問題に取り組む方が興味深い | Yes |
| 25 | I enjoy the process of working with a complex clinical problem and making it more manageable | 私は、複雑な臨床問題に取り組み、それをより扱いやすくする過程を楽しむ | Yes |
| 26 | A good job is one where what is to be done and how it is to be done are always clear* | 良い仕事とは、何をすべきか、どのように行うかが常に明確なものである* | No |
| 27 | To me, medicine is black and white* | 私にとって、医学は白黒はっきりしている* | Yes |
| 28 | The beauty of medicine is that it’s always evolving and changing | 医学の素晴らしさは、常に進化し変化していることである | No |
| 29 | I would be comfortable to acknowledge the limits of my medical knowledge to patients | 私は、患者に対して自分の医学知識の限界を安心して認めるだろう | No |

* These were reverse items.
